# Heavy metal pollution in the aquatic environment: efficient and low-cost removal approaches to eliminate their toxicity: a review

**DOI:** 10.1039/d3ra00723e

**Published:** 2023-06-12

**Authors:** Kosar Hikmat Hama Aziz, Fryad S. Mustafa, Khalid M. Omer, Sarkawt Hama, Rebaz Fayaq Hamarawf, Kaiwan Othman Rahman

**Affiliations:** a Department of Chemistry, College of Science, University of Sulaimani Qlyasan Street Sulaimani City 46001 Kurdistan Region Iraq kosar.hamaaziz@univsul.edu.iq; b Medical Laboratory Analysis Department, College of health sciences, Cihan University-Sulaimaniya Sulaimaniya 46001 Kurdistan region Iraq; c Razga Company Sulaimani City 46001 Kurdistan Region Iraq

## Abstract

Heavy metal contamination of water sources has emerged as a major global environmental concern, threatening both aquatic ecosystems and human health. Heavy metal pollution in the aquatic environment is on the rise due to industrialization, climate change, and urbanization. Sources of pollution include mining waste, landfill leachates, municipal and industrial wastewater, urban runoff, and natural phenomena such as volcanic eruptions, weathering, and rock abrasion. Heavy metal ions are toxic, potentially carcinogenic, and can bioaccumulate in biological systems. Heavy metals can cause harm to various organs, including the neurological system, liver, lungs, kidneys, stomach, skin, and reproductive systems, even at low exposure levels. Efforts to find efficient methods to remove heavy metals from wastewater have increased in recent years. Although some approaches can effectively remove heavy metal contaminants, their high preparation and usage costs may limit their practical applications. Many review articles have been published on the toxicity and treatment methods for removing heavy metals from wastewater. This review focuses on the main sources of heavy metal pollution, their biological and chemical transformation, toxicological impacts on the environment, and harmful effects on the ecosystem. It also examines recent advances in cost-effective and efficient techniques for removing heavy metals from wastewater, such as physicochemical adsorption using biochar and natural zeolite ion exchangers, as well as decomposition of heavy metal complexes through advanced oxidation processes (AOPs). Finally, the advantages, practical applications, and future potential of these techniques are discussed, along with any challenges and limitations that must be considered.

## Abstract 2

An overview of the sources of heavy metal pollution, the related health risks, and cost-effective methods for removing them from wastewater.

## Graphical abstract

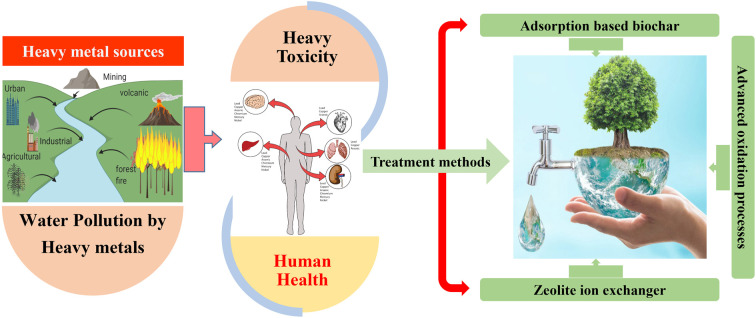

## 1 Introduction

The proliferation of heavy metal ions, which possess toxic properties, in water bodies has emerged as a significant global concern in recent years, owing to the exponential growth in industrialization, urbanization, and the utilization of chemical compounds in various industries.^1^ These substances can have far-reaching and detrimental effects on both the environment and living organisms and are therefore a significant concern for environmental protection.^2,3^ Wastewater effluents from industrial processes are contaminated with a wide variety of toxic heavy metal contaminants, with human and anthropogenic factors being the main causes of increased environmental toxicity.^4^ Heavy metals can be naturally introduced into the environment through processes such as wind erosion of soil, forest fires, volcanic eruptions, biogenic processes, and the release of marine salt.^5^ Anthropogenic contamination of the environment by heavy metals can occur through various means, such as mining operations, the utilization of fertilizers, herbicides, and pesticides, and the irrigation of agricultural land with untreated sewage and industrial effluent.^5,6^ For example, mercury, is introduced into the environment through various human activities, including but not limited to, industries such as chlorine and caustic soda production, paper and pulp preservation, agricultural practices, and the production of pharmaceuticals. Cadmium, another heavy metal, is prevalent in various geological materials such as mineral fertilizers, coal, soils, and rocks. It is also extensively utilized in electroplating processes for diverse purposes including the manufacture of batteries, pigments, textiles, and metal coatings. These activities inevitably lead to an increase in heavy metal contamination in the environment.^5,7^

The toxicity, non-biodegradability, biological accumulation and carcinogenic nature of heavy metals, which pose a significant threat to both the aquatic ecosystem and human health, make their global presence in water a major environmental concern.^8^ Heavy metals, unlike organic pollutants, are not biodegradable and tend to accumulate in living organisms when they are released into the environment, which can negatively impact the health of all forms of life, including humans, animals, and plants.^4,9,10^ Therefore, it is crucial to remove heavy metals in water to mitigate their detrimental impacts on the environment.

A variety of methods and techniques have been developed and implemented to remove heavy metals from wastewater, including physical, chemical, and biological processes. However, after the initial treatment, there is still a need for subsequent treatment methods to further reduce the concentration of heavy metals to safe levels. Adsorption, ion exchange, and membrane technology are examples of physical methods.^11–13^ Chemical methods such as electrokinetic technology, chemical precipitation, and precipitation,^14,15^ as well as biological approaches like phytoremediation and biochar,^16,17^ have been utilized to remove heavy metal ions. Membrane filtration is a process that involves the use of a semipermeable membrane to separate contaminants from water. Membrane filtration methods such as reverse osmosis and nanofiltration have been used for heavy metal removal in many reports.^18–20^ Electrochemical treatment involves the use of an electrical current to induce chemical reactions, resulting in the removal of heavy metals from wastewater. The electrochemical treatment methods such as electrocoagulation, electrooxidation, and electroflotation have been used for heavy metal removal.^21,22^ However, it is worth noting that these methods have certain limitations, for instance, membrane technology can be costly, certain chemical techniques generate significant amounts of sludge, and phytoremediation necessitates extensive monitoring and can be time-consuming. On the other hand, the adsorption method and natural ion-exchanger offer advantages such as affordability, effectiveness, and lack of sludge production.

Extensive research and development have been conducted on the utilization of Advanced Oxidation Processes (AOPs) for the purification of a wide range of waste waters. AOPs can be described as oxidative techniques that utilize an energy input (such as chemical, light, or electrical) to produce reactive oxidizing species (ROS) within the water environment, thereby enabling the degradation of pollutants. Hydroxyl radicals with oxidizing potential of 2.80 V is the primary ROS which have been successfully applied for wastewater treatment to degrade a variety of contaminants such as inorganic complex and recalcitrant organic compounds.^23,24^ AOPs employ a variety of methods such as ozone, hydrogen peroxide, electrical discharge, persulfates and oxygen in different combinations^25,26^ to generate reactive radical species, primarily non-selective hydroxyl radicals. These methods are often used in conjunction with UV-vis irradiation and various types of homogeneous and heterogeneous catalysts to enhance their effectiveness.

Several review articles about heavy metal contamination, toxicity, and remediation strategies have been published in the scientific literature. For instance, Vardhan *et al.*^27^ reviewed “removal of toxic metals (copper, cadmium and zinc) from aquatic system” by conventional treatment methods and suggested the development and improvements of low cost adsorbents for heavy metal removal. Carolin *et al.* analyzed the critical problems and health impacts associated with heavy metals and highlighted the advantages, disadvantages, and limitations of common treatment approaches to identify the reliable technique for heavy metal removal.^28^ Bilal *et al.* presented similar review on “low cost adsorbents” for the uptake of heavy metals from water using bio-waste-based adsorbents.^29^ The author suggested that the future efforts should concentrate on addressing issues such as insufficient removal of heavy metal contaminants, high operational and maintenance expenses, high energy needs, poorer efficiency, and the regeneration of adsorbents for further treatments. A review of the multicomponent adsorption of heavy metals from complex mixtures such as binary, ternary, quaternary, and quinary solutions utilizing various adsorbents is presented in.^30^ The review indicates that adsorbents made from locally and naturally occurring materials such as biomass, feedstocks, and industrial and agricultural waste are effective and promising in removing heavy metals from complex water systems. Nanofiber architectures based on metal–organic frameworks (MOFs) have demonstrated excellent potential for the removal of heavy metal ions from contaminated water. MOFs offer high porosity, remarkable physical and chemical properties, and a high specific surface area, making them effective materials for this application.^31^ However, the high cost and complexity of the preparation process currently limit their widespread use in industrial applications.

**Fig. 1:**
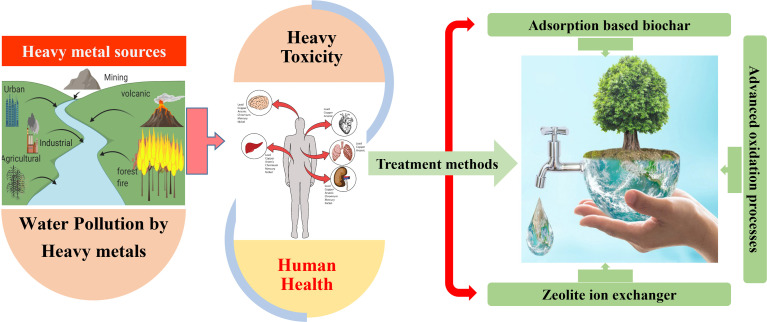
An overview of the sources of heavy metal pollution, their associated health risks, and low-cost, effective methods for removing heavy metals from wastewater.

This article provides an overview of the recent developments and applications of three cost-effective methods for removing toxic heavy metal ions from wastewater. These methods include the biochar-based adsorption approach, the zeolite ion-exchange method, and various AOPs (Fig. 1). In addition, the mechanisms and characteristics of each method are thoroughly discussed. Furthermore, the primary sources of heavy metal pollution in aquatic environments and the associated health risks from heavy metal accumulation and toxicity are also presented. The prospects and limitations for the application of these technologies for the removing heavy metals from wastewater are also emphasized. The current state of remediation processes for removing heavy metal ions from wastewater, including recent advancements and the utilization of adsorption-based biochar, zeolite ion exchanger, and AOPs, is presented along with their advantages and disadvantages.

## 2 Source and toxicity of heavy metals in the aquatic environment

### 2.1 Source of heavy metals

**Fig. 2:**
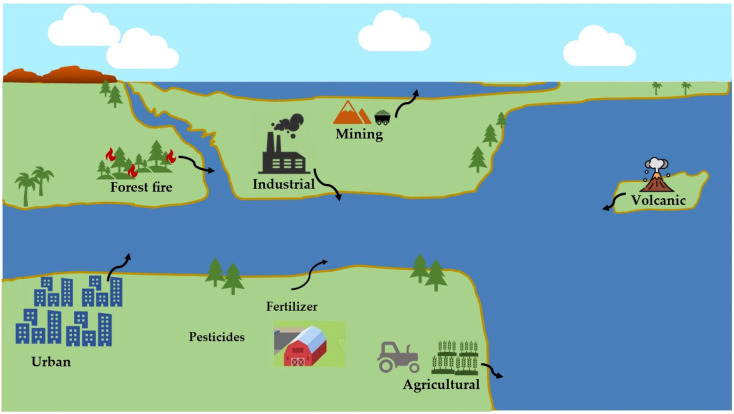
Sources of heavy metals water pollution.

The primary source of heavy metal contamination in the environment has been determined to be the exponential increase in human population, the proliferation of industrialization, and the expansion of agricultural activities.^32^ Heavy metals, known for their high toxicity, long-lasting presence, and bioaccumulation, have a significant effect on the quality and safety of water.^33^ Heavy metals are a group of elements characterized by their high density and atomic weight that can be detrimental to both human health and environment. Heavy metals have become a significant problem for water pollution in recent times, raising public concern for the environment and human health, and are considered a major global concern currently.^34,35^ Therefore, understanding the sources, chemical transformations, leaching processes, and modes of deposition of heavy metals are necessary to mitigate the risk they pose to the environment and human health. In this section, an overview of the worldwide situation of heavy metal contamination in water bodies is presented, with a specific focus on the examination of various anthropogenic and natural sources (Fig. 2). The introduction of heavy metals into the environment can be attributed to both anthropogenic and natural causes. Human activities such as industrial operations, mining, irrigation of crop fields with industrial water, and industrialization and agricultural practices contribute significantly to heavy metal pollution,^36^ while natural phenomena such as volcanic eruptions, weathering of rocks, biogenic processes, and wildfires also contribute to the introduction of heavy metals into the environment.^5^ The release of significant quantities of wastewater containing harmful heavy metals into the environment is primarily caused by modern industrial processes like electroplating, production of electronic devices, mining, metallurgy, smelting, fertilizer production, nuclear fuel, paper manufacturing, power plant emissions, and chemical etching.^7^ Industrial activities are known to release heavy metal-contaminated wastewater into the environment, either through direct discharge into water bodies or through leakage or runoff from industrial sites, resulting in severe water pollution.^5^ Research has shown that agricultural and industrial activities that do not have a single identifiable source, known as non-point source pollution, are major contributors to the presence of cadmium, nickel, lead, zinc, arsenic, and mercury in the environment. Additionally, significant amounts of heavy metals can also originate from natural sources such as atmospheric deposits, which can be transported to the surface of the earth through precipitation. The aforementioned sources are the main causes for polluting aquatic environment by heavy metals.^37–39^ Unless proper measures are taken to control them, there is a danger of anthropogenic wastes releasing heavy metals into water sources, with potentially serious implications for human health and aquatic life.^40^ Previous research has indicated that the level of heavy metal contamination in aquatic systems fluctuates seasonally due to changes in precipitation and human behavior.^41,42^ For example, the World Health Organization (WHO) has established a maximum allowable limit of 10 μg L^−1^ for arsenic in drinking water. However, a review of the literature has revealed that many countries, including Bangladesh, Iran, Pakistan, Mexico, Saudi Arabia, China (in the Yangtze River and Han River basins), Latin America, the USA, and Ethiopia, have reported arsenic concentrations exceeding this permissible limit. The risk of exposure to arsenic-contaminated drinking water is highest in Asia.^43^ Therefore, it is crucial to conduct an in-depth investigation into the dynamic changes and sources of heavy metal pollution resulting from the expansion of aquatic products, especially in regions where drinking water quality is compromised. This kind of research can help to identify the main contributors of heavy metal contamination and provide a better understanding of the environmental impacts of aquatic product expansion. Additionally, it can facilitate the development of effective strategies and policies to manage and mitigate the adverse effects of heavy metal pollution on aquatic ecosystems and public health.

### 2.2 Toxicity and effects of heavy metals on the environment and human health

**Fig. 3:**
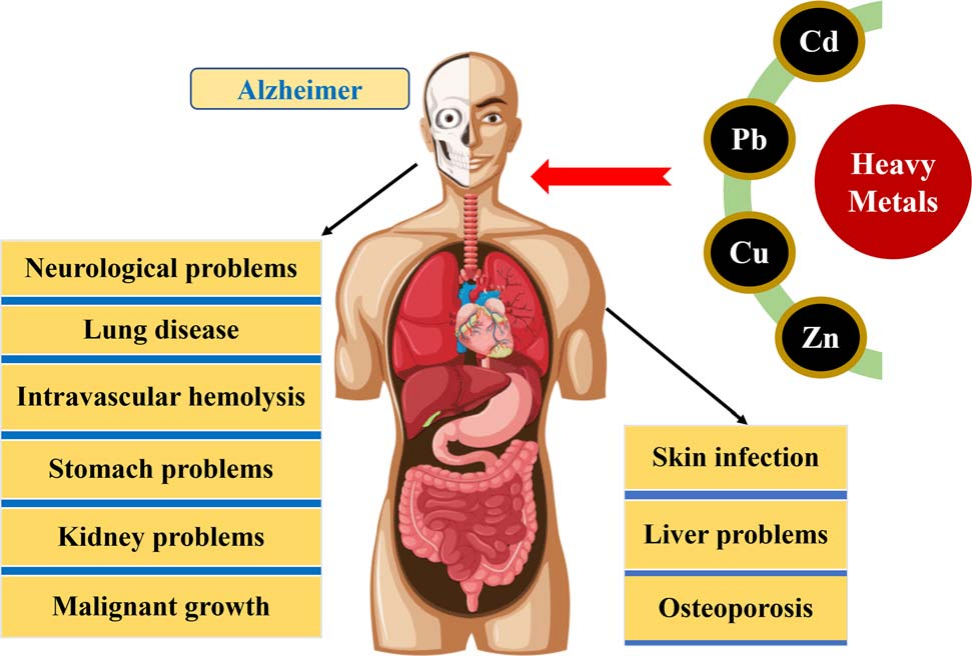
Impacts of heavy metals toxicity on human health.

The environmental persistence and irreversible biotoxicity are the characteristics of heavy metals like arsenic, cadmium, lead, and copper. Chronic exposure to heavy metals at low concentrations can have negative effects on the environment and the human body, including teratogenic and carcinogenic effects.^44,45^ Water contaminated with heavy metals poses a danger to all types of ecosystems and the human of health and living organisms, as these elements do not degrade in the environment and can build up in living tissue, disrupting the food chain.^46^ Although certain heavy metals are necessary for physiological processes and serve as enzyme co-factors, micronutrients, osmotic pressure regulators, and molecule stabilizers in living organisms, most heavy metals do not have any known biological function and can be toxic when present in excess.^47^ Additionally, certain heavy metals like Cd and Pb, even in trace amounts, pose a significant risk to human health.^48^ The level of harm caused by heavy metals in the environment depends on the bioavailability, which is the proportion of the metal that can be taken up by living beings and the dose absorbed by them. The presence of heavy metal ions in the environment is natural, however, their concentration is rising with the increase of industrial waste. Potential health hazards can occur as a result of harmful heavy metal ions entering the food chain and accumulating in the human body.^46^ Heavy metals can enter the human body through various routes such as absorption, skin contact and inhalation, resulting in a range of health issues from mild to severe, including loose intestines, anxiety, lung disease, fatigue, kidney problems, stomach issues, skin infections, neurological issues and malignant growth (Fig. 3). Some of these health issues are caused by acute toxicity, while others are caused by chronic exposure to low levels of heavy metals.^49^ Aquaculture ponds have been found to have water eutrophication and heavy metal pollution, caused by the use of improper aquaculture methods, some of which are even more severe than safety standards allow.^50,51^ The high levels of heavy metals, by contaminating the food chain, can cause severe ecotoxic stress on humans and aquatic organisms.^52^ Therefore, given the varying nature of heavy metal pollution in aquaculture environments, it is crucial to understand how the ecological risks of heavy metal exposure change during the growth period of aquatic products.

Soils contaminated with heavy metals restrict the growth and survival of plants, leading to nutritional, ecological and evolutionary challenges.^53^ Factors such as plant species, concentration, type of metal, chemical form, and the composition and pH of the soil affect the toxicity of heavy metals in plants. The presence of heavy metals can alter the diversity, quantity, and function of microbial populations, as well as the genetic makeup of the microorganisms. Heavy metal toxicity can alter the structure of nucleic acids, disrupt cell membranes, interfere with cellular functions, inhibit enzyme activity, and affect energy production, leading to lipid oxidation and protein damage, altering the morphology, metabolism and growth of microorganisms.^54^ Exposure to heavy metals through food can lead to accumulation in human bones and fatty tissues, leading to nutritional deficiencies and weakened immune responses. Cadmium and lead, among other heavy metals, have been found to be associated with intrauterine growth retardation.^55^ In history, numerous instances of food contamination caused by industrial pollutants have been extensively recorded. Several nations, including Japan, Iraq, and the United States, have encountered incidents where thousands of people fell sick or died. Among these, the Minamata disease, a case of methylmercury poisoning, is the most well-known. The outbreak was first discovered in 1956 around Minamata Bay in Kumamoto Prefecture, Japan, and a second epidemic occurred in 1965 along the Agano River, in Niigata Prefecture, Japan.^56^ Symptoms included sensory disturbance, cerebellar ataxia, hearing and speech issues, and narrowing of the visual field. Consumption of contaminated fish and shellfish, which accumulated the discharged methylmercury, caused the poisoning. In the Jinzu river basin of Japan, before 1960, the local population suffered from an endemic illness known as “itai–itai” due to the consumption of rice contaminated with a high level of cadmium. Mitsui Mining and Smelting's Kamioka Mining Station was identified as the source of the cadmium pollution after an investigation in 1961. The worst-affected areas were 30 km downstream of the mine. It was not until 1968 that the Ministry of Health and Welfare of Japan officially announced that the symptoms of “itai–itai” disease were caused by cadmium poisoning.^57^

## 3 Effective methods for heavy metal remediation

The natural surface water environment, being the most prevalent freshwater ecosystem, has been the subject of significant research efforts towards the risk assessment and monitoring of heavy metal pollution. As a result, several research studies have shown the presence of heavy metal pollutants in a variety of water systems, including surface water, seawater, wastewater, and even groundwater.^46,58^ Heavy metal pollution removal from wastewater systems has received considerable attention recently and developing an efficient and eco-friendly removal method requires extensive study. The elimination of heavy metal ions from point sources is necessary to protect the environment due to their toxicity, bioaccumulation potential, and persistence in nature.^59^ In the literature, several remediation techniques have been proposed for the treatment of contaminated water by heavy metals.^60^ These techniques include ion exchange, advanced oxidation processes, chemical precipitation, electrochemical processes, adsorption, reverse osmosis, solvent extraction, and membrane filtration, which have been shown to be effective in previous studies.^28,61–63^ In addition to these techniques, other remediation methods have been explored, including phytoremediation and chemical techniques such as chemical precipitation and electrokinetic technology^48,49,64–66^ as well as physical methods such as adsorption,^67,68^ ion exchange,^69,70^ and membrane filtration.^71^

New acid and amino functionalization approach using a Zr-based molecular organic framework was developed and applied for selectively removing heavy metals from wastewater.^72^ MXenes, a new group of 2D materials, offer a broad range of possibilities for water and effluent treatment owing to their unique characteristics and beneficial applications. These properties include high sorption–reduction capacity, superior electrical conductivity, hydrophilicity, and increased thermal stability. The exceptional sorption selectivity of MXenes makes them an ideal choice for eliminating hazardous heavy metal pollutants.^73^ Vacuum distillation is a highly effective technique for removing metals from aqueous solutions, recovering metals, and impregnating metals into porous materials. The process involves heating the solution to a high temperature under reduced pressure, which causes the metals to vaporize and separate from the solution. The vapor is then condensed and collected, leaving behind a purified solution. This method has several advantages, including the ability to selectively remove specific metals from the solution and the potential to recover and reuse the extracted metals. Additionally, vacuum distillation can be used to impregnate metals into porous materials, which can improve the materials' mechanical, thermal, and electrical properties.^74^ While chemical methods have been demonstrated to be efficient in the removal of heavy metals, it is important to note that these methods also produce substantial quantities of sludge and incurs significant costs in terms of energy and economic expenditure. Phytoremediation an environmentally friendly method with minimal byproduct production, but it is a slow process and requires careful attention and monitoring.^75^Table 1 presents a comprehensive overview of the main benefits and limitations associated with both classical and contemporary technologies used for the removal of heavy metals from wastewater.

**Table 1:** Comparison of various remediation methods for heavy metals removal from water

Methods	Strengths	Limitations	Ref.
Photocatalysis	Possess high oxidizing potential, can degrade heavy metal complexes, produce no sludge, and capable in degradation of organic complexing agents	Costly investment for equipment	80
Phytoremediation	Environmentally benign	It is a slow process and necessitates a large amount of land	81
Chemical precipitation	Low-cost and simple method that effectively removes most heavy metals	The sludge generation, expenses to its management, and usage of chemicals	82
Reverse osmosis	Easily operable, chemical-free, and compatible with other methods	Energy-intensive, costly equipment and operations, membrane fouling and poor water permeability	83
Electrochemical treatment	Effective metal recycling technology with minimal chemical usage	Operational costs, and lack of efficiency, stability, and selectivity	84
Flotation	Low-cost and dewatering	High operating and maintenance costs	85
Coagulation/Floculation	Easy separation of the resulting products and pollutant absorption	Generation of secondary pollution	86
Membrane filtration	Efficient separation and selective at low pressure	Expensive operation, scaling, and fouling	87
Adsorption	Applicable in wide pH range, high removal efficiency, ease of use, flexibility, cost-effective, and simplicity in design	Regeneration of the adsorbent is necessary	88
Ion-exchange	Low-energy regeneration of resin and economical	Resin fouling and adsorption of organic substances	89
AOPs	Formation of *in situ* reactive radicals, minimal or no chemical usage, and no sludge generation	Not applicable in large-scale	90

In certain instances, it may be necessary to employ a combination of removal technologies to treat complex wastewater contaminated with heavy metals. In recent years, numerous studies have been conducted which have successfully examined the removal of heavy metals and organic pollutants that coexist in wastewater.^76–78^ The selection of an appropriate treatment method depends on a variety of factors, including the economic and environmental impacts associated with the method. Therefore, it is important to carefully evaluate and select the most appropriate combination of removal technologies for each specific situation to achieve efficient and effective removal of heavy metals and organic pollutants from wastewater. In recent years, a new class of nano adsorbents with distinct properties has emerged and been utilized in the treatment of wastewater. These nano adsorbents possess features such as a significantly increased surface area, excellent chemical stability, green and reusable materials, among others. Furthermore, there have been notable advancements in the use of Metal–Organic Frameworks (MOFs) for the removal of hazardous metals such as mercury (Hg), lead (Pb), chromium(vi), cadmium (Cd), and arsenic (As) from wastewater. In addition, MOFs have also demonstrated effective removal of organic dyes such as methyl red, rhodamine B, congo red, reactive black, methylene blue, methyl orange, among others. These recent developments have been well-documented in ref. 79

Among the various treatment technologies available for the removal of heavy metal complexes from wastewater, the utilization of natural zeolite ion exchangers, adsorption based on biomass-derived biochar, and AOPs have emerged as the most practical options due to their exceptional characteristics such as cost-effectiveness, high efficiency, and ease of operation. The utilization of natural zeolites as ion exchangers has been found to be effective in removing heavy metals such as copper, cadmium, and lead from wastewater due to their high selectivity, high cation exchange capacity, and low cost. Similarly, the use of biomass-derived biochar as an adsorbent has gained significant attention in recent years due to its low cost, abundant availability, and high adsorption capacity for heavy metal ions.^91^ AOPs such as Fenton, photo-Fenton, and electro-Fenton have also been shown to be effective in removing heavy metal complexes from wastewater due to their ability to generate highly reactive oxidizing species, which can degrade complex organic pollutants and release free heavy metal ions that can be eliminated using conventional treatment methods.^92^

On the other hand, the use of natural soil and mineral deposits to remove heavy metals from wastewater appears to be the least effective method, as their adsorption capacity is limited and often requires large amounts of material for efficient removal. Therefore, the selection of the appropriate treatment technology for heavy metal removal from wastewater depends on several factors such as the type and concentration of heavy metals, the characteristics of the wastewater, and the economic and environmental feasibility of the treatment method. Heavy metal ions have the ability to form stable complexes with different organic compounds found in wastewater, such as citrate, humic acid substances, and other ligands, which can result in varying structures and toxicity levels.^93^ AOPs produce hydroxyl radicals, which can break down the chelated metals, thereby releasing the free metal ions or organic matter, leading to an enhanced removal efficiency. This work provides an up-to-date review of zeolite ion exchangers, adsorption-based biochar, and AOPs for removing heavy metals from water, with an emphasis on important performance indicators and commercialization prospects. Additionally, this article will identify the limitations in previous and current research on the removal of heavy metal ions, in order to facilitate the design of experimental procedures that address these shortcomings for each individual process.

### 3.1 Adsorption-based biochar

Adsorption is one of the most commonly utilized method that has demonstrated its effectiveness and economic viability for the removal of heavy metal ions from contaminated water. The adsorption process has emerged as the principal method for the removal of heavy metals from wastewater due to its efficiency, ease of implementation, and adaptability with regards to the operation, design, and environmental considerations. This section discusses the utilization of various raw materials as biomass feedstocks for the laboratory-scale production of biochar adsorbents and provides information on the efficacy of synthesized biochar for removing common heavy metal ions from wastewater. Numerous studies are published annually to investigate the technical performance of various adsorbents and their composites in terms of adsorption capacity, production techniques, and regeneration application. The primary advantages of adsorption-based treatment technologies are the minimal generation of residual waste and the capability of recovering and reusing the adsorbents.^94^ A variety of adsorbents have been employed in the removal of heavy metals, such as activated carbon, zeolites, alumina, manganese oxide, and iron oxides.^95–97^ Recently, it was found that removing heavy metal ions from wastewater using a porous composite hydrogel based on graphene proved effective.^98^ Both agricultural waste materials, such as walnut shells, coffee grounds, rice husk ash and sawdust,^99–101^ and industrial waste materials, such as red sludge, fly ash from power plants, and steel slag, have been utilized as adsorbents for heavy metal ions removal from water.^99,102,103^ The utilization of biochar as an adsorbent has demonstrated significant potential in the purification of water, particularly for treating heavy metal pollutants, pesticides, and other deleterious contaminants.

Over the period from 1999 to 2020, significant advances have been made in biochar research, which can be classified into three distinct phases: initial budding (before 2009), Primary growth (2009–2015), and rapid development (after 2015). Following 2016, there has been a noticeable shift in research trends, with a more diverse range of publications and a greater depth of research content.^104^ Biochar is a carbon-rich substance produced as a byproduct of the pyrolysis of biomass at temperatures ranging between 250–800 °C in oxygen-limited or oxygen-free condition.^105,106^ This inexpensive substance, that addresses sustainability issues through carbon sequestration has been utilized in various fields of application, including crop productivity, soil enhancement, wastewater treatment by adsorbing harmful heavy metal contaminants. The composition of biochar plays a crucial role in determining its potential use.^107,108^ Several studies have reported that biochar can serve as an excellent photocatalyst and mediator for AOPs. The numerous functional groups on the surface of biochar allow the production of Persistent Free Radicals (PFRs) and ˙OH, which can decompose organic pollutants. Biochar is a widely accepted adsorbent due to its low cost, pore filling effect, π–π stacking interaction, and hydrogen bonding. Additionally, activated, and modified biochar has been used as a potent agent for removing various organic pollutants, including organic dyes and antibiotics.^109,110^ Biochar-based adsorption has been demonstrated to be an effective method of eco-remediation in reducing heavy metal pollution in water and soil, with multitude of advantages. Extensive research has been conducted on the utilization of adsorbents, specifically biomass-based activated carbon compounds and biochar derived from agricultural waste, owing to their exceptional capacity to remove heavy metal pollutants present in wastewater.^55,111^ Biochar has been widely studied due to its high specific surface area and pore volume, a wide range of functional groups, ability to synthesize from various raw materials, and eco-friendly nature, has been widely considered as an efficient adsorbent and has the potential to be a valuable tool for the removal of harmful contaminants.^112^ The physico-chemical properties of biochar can vary depending on the various physical and chemical modification techniques employed, which can greatly enhance the efficiency of heavy metal ion removal from wastewater. The adsorption of heavy metals by biochar is influenced by various parameters such as the dose of biochar, water temperature, pH of water, type of heavy metals, characteristics of biochar, initial concentration, and the presence of other cations in the water.^113^ The shoot and root of biochar derived from *Plumbago zeylanica* have been found to exhibit satisfactory efficiency for removing chromium and cadmium under neutral conditions.^3^

Biochar is created through the utilization of various raw materials, comprising of a wide range of solid waste, agricultural waste, and sewage treatment plant sludge, including but not limited to crop straw, orange peel, and animal manure.^114^ Biochar is abundant in oxygen functionalities, specifically carboxylic acid, phenolic, and sulfonic groups, which constitute 27–34% of its composition. These oxygen functional groups have been found to increase the catalytic activity and molecular absorption capacity of the biochar surface, making it a promising adsorbent material for the removal of heavy metal ions.^115^ The choosing appropriate feedstock is crucial in determining the properties of biochar, as different feedstocks can lead to variations in biochar properties. Therefore, it is imperative to identify the appropriate feedstock to ensure that the generated biochar possesses the desired characteristics. Lignocellulosic biomass has been the subject of extensive research, and is widely employed as feedstock for thermochemical processing to yield high-quality charcoal and bio-based fuel with minimal ash content.^116^ The conversion of ragweed and horseweed into biochar through unmodified direct pyrolysis was found to be highly efficient in the removal of Cd(ii) and Pb(ii) from aqueous solutions.^117^ Experimental studies have demonstrated that unmodified biochar has a limited adsorption capacity. Therefore, modification is often necessary to enhance its adsorption performance. In recent years, researchers have developed various biochar modification strategies, which have been extensively used as an efficient and environmentally friendly adsorbent due to their superior specific surface area, porous structure, and surface functional groups when compared to the pristine biochar.^63^ Researchers have adopted two different strategies for modification of biochar: direct modification of the biomass feedstock and modification of existing biochar.^118^ Biochar modifications increase various oxygen-containing functional groups (such as –OH, –COOH, –O–, CO–, *etc.*), which increases the number of active sites for heavy metal ions, thus enhancing the biochar's adsorption ability.^115^Table 2 demonstrates the efficacy of utilizing biochar derived from different biomass feedstocks for removing heavy metal ions from water and wastewater, both before and after modification. Previous studies have demonstrated that the ability of biochar to adsorb and selectively target specific heavy metal ions is greatly impacted by its physical and chemical properties, such as surface area, pore structure, chemical composition, functional groups present on the surface, capacity for ion exchange, and pH level, which can vary depending on the source material and pyrolysis conditions used in its production. The surface of biochar is composed of various functional groups, such as hydroxyl, carboxylic, carbonyl, and amino groups, that exhibit high binding potential for heavy metal ions. Additionally, the surface of biochar contains various inorganic constituents that have been known to enhance the adsorption capabilities, resulting in the complexation and co-precipitation between biochar and heavy metal ions. The cation exchange capacity of biochar is enhanced by the presence of negative charges on its surface. This creates an electrostatic attraction between biochar and positively charged heavy metal ions, leading to superior adsorption capabilities.^119^

**Table 2:** Heavy metal removal from water using biochar derived from different feedstocks

Heavy metals	(mg L^−1^)	Biomass feedstocks	% Removal	Modified by	% Removal	Ref.
Cu(ii)	50	Corn straw	42.3%	α-FeOOH	71.9%	120
Cu(ii)	40–300	Coconut shell	32%	Fe_3_O_4_-alginate	96%	121
Cu(ii)	10–300	Sawdust	70%	Amino groups	5 to 8 folds	122
Cd(ii)	33	Paper mill sludge	90%	Under CO_2_	90%	123
Cd(ii)	20	Rice straw	90%	Fe_3_O_4_	100%	124
Cd(ii)	30	Rice husk	80%	MgO-modified	100%	125
Cr(vi)	50	Poplar	100%	Fe-modified	3.8 folds	126
Pb(ii)	400	Crofton weed	100	MgO	5 folds	127
Pb(ii)	150	Swine sludge	100	Thiourea	5–8 folds	128
Pb(ii)	50	Sewage sludge	100	KOH and CH_3_COOK	5 folds	129
As(v)	4	Cotton stalks	81–98%	H_3_PO_4_ and KOH	90–99.5%	130
As(v)	10	Corn straw	80	Fe-impregnated	400 fold	131
As(iii)	10	Rice straw	90	Fe_3_O_4_	100%	124

**Fig. 4:**
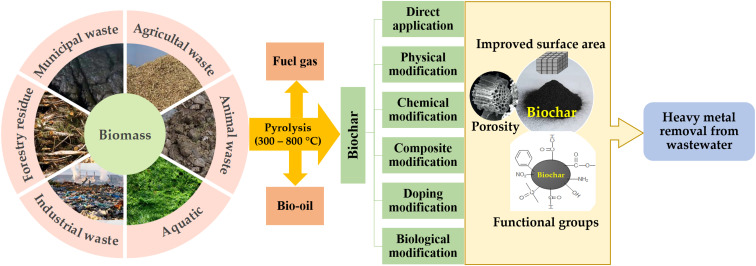
Methods of production and modification of biochar from various biomass feedstocks for absorbing heavy metals present in wastewater.

The chemical composition of biochar is directly related to the feedstock used in its production, as different raw materials result in variations in the constituent elements present in the final product. The feedstock used in biochar production have a significant impact on its elemental composition with variations in the proportions of cellulose, hemicellulose, and lignin present different biomass sources. Biochar primarily consists of carbon, hydrogen, oxygen, nitrogen, and various trace elements. Biochar derived from animal-based materials typically containing higher concentrations of trace elements, such as phosphorus, magnesium, calcium, and potassium, when compared to plant-based biochar.^132^ The pyrolysis temperature plays a significant role in determining biochar properties. Higher temperatures lead to a decrease in the presence of elements such as hydrogen, nitrogen, sulfur, and others, as well as a decrease in cation exchange capacity and the number of oxygen-containing functional groups on the surface, conversely, it also leads to an increase in the degree of aromaticity in the biochar. On the other side, an increase in pyrolysis temperature leads to an enhancement in the specific surface area, pore structure, and pH level of biochar, making it more effective for the adsorption of heavy metals.^63^ The capability of biochar in heavy metal ion adsorption is contingent upon its characteristics, which can be affected by various factors including the feedstock utilized, production method, thermal decomposition temperature, duration of heating, pretreatments procedures, and modification methods. Fig. 4 illustrates the various methods used for producing and modifying biochar.

Recent studies have primarily concentrated on modifying biochar to enhance its surface characteristics and structure for improving environmental benefits and remediation effectiveness, including increasing its pore size, surface area, and surface functional groups. The modification of biochar can be achieved through various techniques such as chemical, physical, and magnetic modifications and impregnation with mineral oxides. Additionally, pretreatment of the raw materials with different chemical agents can also be used to modify the properties of the biochar. Acid–base modification can significantly enhance the physicochemical properties of biochar.^133^ Treatment of biochar by nitric acid results in the expansion of micropores into macropores through the breakdown of pore walls, leading to an increased presence of acidic functional groups such as hydroxyl, ketonic, carboxylic, and other oxygen-containing moieties.^134^ Alkaline treatment, on the other hands, results in a biochar with greater surface area, a higher surface aromaticity, and a higher ratio of nitrogen to carbon, but a lower ratio of oxygen to carbon.^135,136^ Chemical oxidation methods, such as the use of potassium permanganate, hydrogen peroxide, sodium persulfate, and ozone, have also been employed to modify the surface functional groups present on biochar.^94,137^ The addition of amino functional groups to the surface of biochar increase its basic properties and can greatly enhance its ability to adsorb and remove heavy metal ions from contaminated water. Researchers have used different methods to modify biochar, such as using chitosan to introduce amine functional groups, hydrogen peroxide to increase oxygen-containing functional groups, and concentrated sulfuric and nitric acids to prepare amino-modified biochar made from bamboo, peanut hulls, and other sources.^138–140^ Many studies have attempted to develop magnetic-modified biochar to enhance the separation efficiency of biochar particles after the wastewater treatment process.^141–145^

One of the challenges in using adsorption-based treatment is its poor selectivity for heavy metals in complex wastewater matrices. The presence of other competing ions, organic matter, or pH fluctuations can affect the adsorption performance of biochar. To improve the selectivity of biochar, some strategies have been proposed, such as using biochar-based nanocomposites, magnetic biochar, or biochar-supported nanoparticles.^146,147^ These methods can introduce new functional groups or magnetic properties to the biochar surface, which can increase the specificity and ease of separation of biochar from wastewater.^148^ Another challenge of biochar is its potential environmental risks after adsorption of heavy metals. The spent biochar may pose a threat to the soil and water quality if not properly disposed of or regenerated. Therefore, some methods have been suggested to reduce the environmental impacts of biochar, such as regeneration, reuse, or safe disposal. Regeneration can restore the adsorption capacity of biochar by removing the adsorbed heavy metals with acids or complexing agents. Reuse can utilize the spent biochar as a fertilizer or soil amendment to enhance the soil fertility and crop productivity. Safe disposal can involve using the spent biochar in construction or electronic industries as a raw material or a component.^146^

Biochar regeneration is an inverse process of adsorption that aims to restore the adsorption capacity of biochar and reduce the environmental impact of the adsorbate. Chemical regeneration is one of the most widely used methods because it can effectively desorb metal ions from biochar and maintain its structure and surface properties.^149^ Chemical regeneration involves using acids, bases, salts, or oxidants to wash or soak the metal-loaded biochar and then separate the metal-rich solution from the regenerated biochar. The effectiveness of chemical regeneration depends on several factors, such as the type and concentration of the chemical agent, the contact time and temperature, the pH and ionic strength of the solution, and the properties of the biochar and metal ions.^149,150^ The advantages of chemical regeneration include high desorption efficiency, simple operation, and low energy consumption. However, some challenges also exist, such as secondary pollution, corrosion, safety issues, and loss of biochar mass and porosity. Therefore, there is still a need for further research and development on the optimal conditions and mechanisms of biochar regeneration for sustainable wastewater treatment.

### 3.2 Removing heavy metal by zeolite ion exchanger

**Fig. 5:**
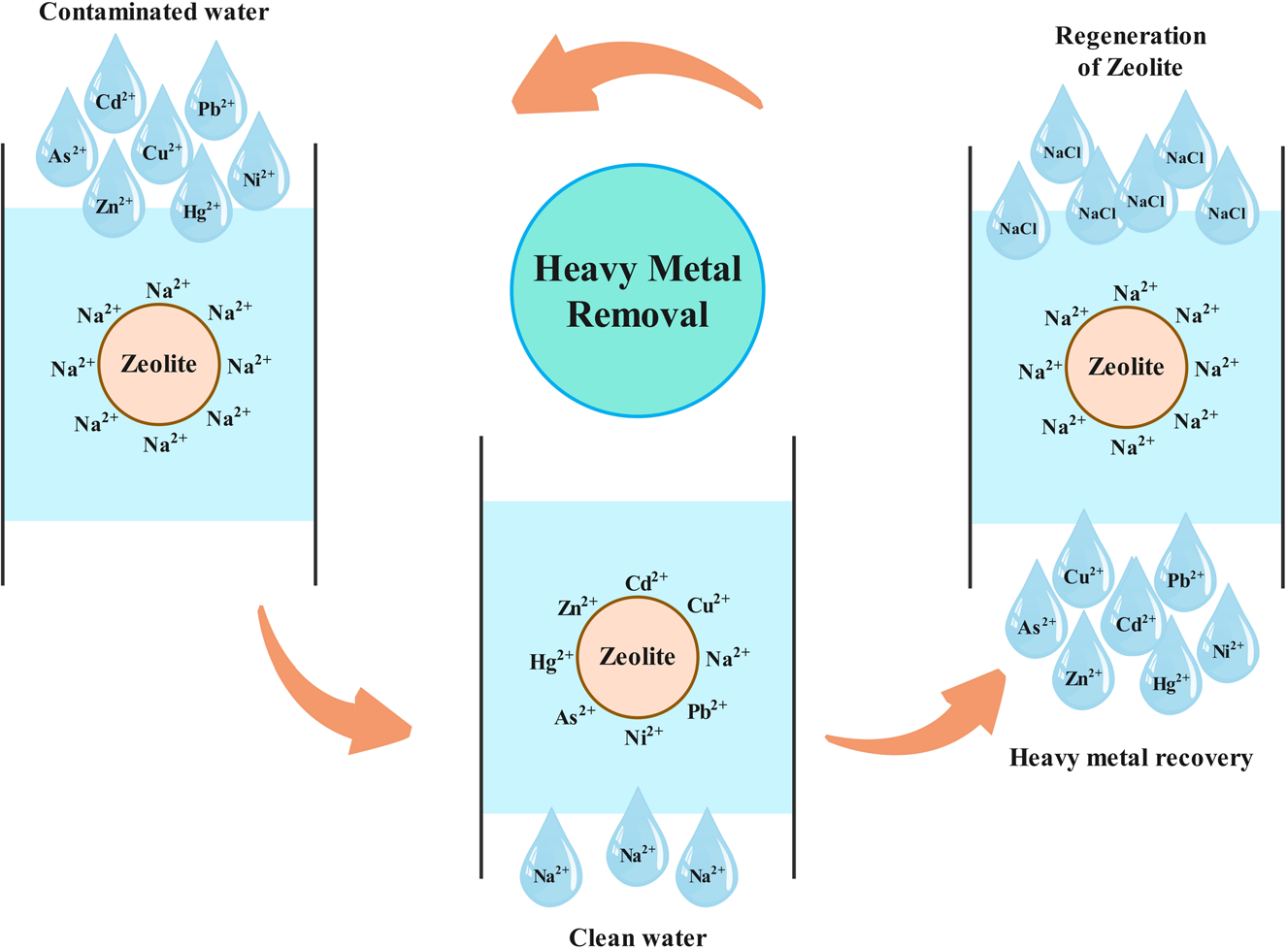
Ion exchange mechanism of removing heavy metal ions in water using zeolite.

The process of removing heavy metal ions from wastewater using ion exchangers is commonly used, particularly in industries that involve metal processing.^60^ The fundamental principle of this method is reversibility of ions. The effectiveness of this method is largely dependent on the formation of functional groups and complexes with counter-ions.^88,151^ Zeolite is a microporous aluminosilicate mineral that is composed of silica (SiO_2_) and alumina (Al_2_O_3_) tetrahedrons and it is characterized by its unique crystal structure, which consists of a hollow polyhedral framework of tetrahedrons at its core. This skeletal structure is highly porous, comprising of numerous uniformly sized cavities or pore channels of molecular size, providing ample space for molecules to be accommodated and exhibit considerable freedom of movement, thereby facilitating ion exchange. Zeolites exhibit distinctive characteristics such as high specific surface areas, ordered and homogenous microscopic pores, interconnected pores, and the capability for regulating molecular interactions. Zeolites are a relatively novel, inexpensive type of inorganic aluminosilicate cementitious material that is simple to employ and resource-efficient. They possess the capability of being tailored to specific applications and are obtainable in both semi-crystalline and amorphous three-dimensional network-bonded structures. Due to their rapidly advancing controlled characteristics, zeolites are increasingly being utilized in a variety of creative applications and are gaining popularity.^152,153^ Zeolites are highly attractive to researchers due to their numerous advantageous properties, such as efficient adsorption characteristics, good recoverability, high thermal stability, a distinct pore structure, exceptional ion exchange abilities, large specific surface area, high porosity, applications in purification, high surface activity, and robust ion exchange capacity. As a result, zeolites are now widely employed in a variety of sectors, such as environmental protection, petrochemicals, and more. The utilization of geopolymer-based zeolites as an adsorbent has been deemed a potentially effective means for the removal of toxic heavy metal ions from wastewater.^153,154^ A study was conducted to examine the adsorption properties of a natural zeolite (clinoptillolite, sourced from Western Anatolia) in order to determine its efficacy for removing Co^2+^, Cu^2+^, Zn^2+^, and Mn^2+^ from wastewater.^91^ The findings of the study indicate that natural zeolite can effectively be utilized as a low-cost and easily accessible material for the removal of heavy metal ions from wastewater. Synthesized zeolite, obtained from lithium leach residue through the hydrothermal method, was successfully applied as an efficient adsorbent for removing lead and cadmium ions from water.^155^ Experimental evaluations have been conducted on natural Jordanian zeolites sourced from Al Mafraq, located in the northeastern region of Jordan, to determine their effectiveness in the removal of cadmium and copper ions from aqueous solutions.^156^ The ability of natural zeolite sourced from the Yagodninsky deposit in the Kamchatka region to remove nickel, copper, cobalt, iron, and their mixtures from aqueous solutions was studied.^157^ A study was conducted on the ability of stable Na-clinoptilolite zeolite in acidic pH medium to remove various heavy metal ions from water, and the zeolite's selectivity on the efficiency of the adsorption process was also evaluated.^158^ In recent years, several review articles have been published for utilization of natural, modified and synthetic zeolites in removing heavy metal ions from water.^88,153,159–165^ The development and the utilization of low-cost resources for the synthesis of zeolites is a promising area of research. Research has demonstrated that a range of valuable zeolite products can be synthesized from waste materials, such as fly ash and blast furnace slag. This approach is aimed at implementing a waste-to-resource strategy for environmental remediation.^166–170^ By using a traditional hydrothermal method, researchers were able to synthesize zeolite NaA from lithium leach residue, a waste material generated in the process of extracting lithium from lithium ore *via* alkaline leaching. This material was found to be an efficient adsorbent in removing lead and cadmium ions from water.^155^ It has been reported that sulfur-modified chabazite, a cost-effective ion exchange resin, can be utilized for the selective removal of strontium and cesium ions.^171^ Additionally, there is ongoing investigation into utilizing other waste materials from various industries for the synthesis and modification of zeolites, with the aim of increasing their adsorption capacity.^170,172–174^ Natural zeolites are highly promising materials for removing heavy metals from various sources that contaminate water. The unique properties of natural zeolites, such as ease of ion exchange, adsorption, dehydration, and rehydration, as well as their eco-friendliness, low cost, regenerability, accessibility, and availability, make them an excellent choice as adsorbents for removing heavy metal ions from wastewater.^175^ Zeolites have exceptional exchange capacity for removing heavy metal ions and can be easily regenerated using low-cost sodium chloride solutions, even under varying operating conditions (Fig. 5). The use of zeolites as a cost-effective alternative with superior ion exchange and absorption properties offers significant potential for the removal of heavy metal ions from wastewater.

### 3.3 Degradation of heavy metal complexes by advanced oxidation processes

The constitution of wastewater containing heavy metals is complicated by the presence of complexing agents including but not limited to pesticides, fertilizers, detergents, plasticizers, pharmaceuticals, oils. Heavy metal ions have the ability to form stable complexes with a diverse range of structures and toxicity by interacting with common complexing agents, such as citrate, ethylenediaminetetraacetic acid (EDTA), nitrilotriacetic acid, cyanide, antibiotics, humic acid substances, and other ligands.^76^ In recent years, heavy metal complexes have garnered significant attention due to their inherently high toxicity and recalcitrant nature.^176^ The difficulty in removing hazardous heavy metals from wastewater stems from their bond formation with the thiol groups of proteins, leading to their persistence in the water in either a combined or chemical state.^177^ Heavy metal complexes, which are prevalent in wastewater originating from modern industries, are found to be more stable and recalcitrant in comparison to free heavy metal ions.^178,179^ The presence of organic ligand chelators, such as nitrilotriacetic acid, EDTA, diethylenetriamine pentaacetate, and citric acid, in water can result in the formation of stable heavy metal complexes through the complexation of heavy metal ions. These complexes are typically resistant to removal by conventional wastewater treatment techniques, such as alkaline precipitation, ion exchange, and adsorption.

**Fig. 6:**
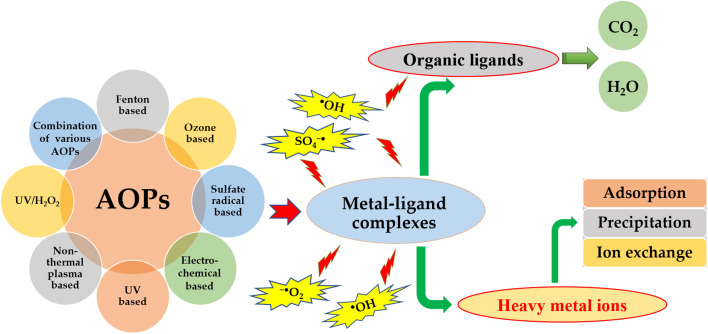
Decomposing heavy metal complexes *via* various AOPs in water.

Advanced oxidation processes (AOPs) have been found to be effective in degrading heavy metal complexes present in wastewater, thereby enhancing the efficiency of metal removal.^180^ AOPs are a group of oxidative treatment techniques that rely on the formation and utilization of highly reactive oxygen species (ROS) to remove various pollutants. These ROS, such as hydroxyl radical (˙OH) and sulfate radical (SO_4_˙^−^), have high oxidation potential and can effectively degrade various organic and inorganic compounds.^181,182^ The utilization of Advanced Oxidation Processes (AOPs) has been shown to be more efficient and effective in comparison to other methods in decomposing heavy metal complexes. The mechanism of AOPs involves the generation of hydroxyl radicals and other reactive species that serve to break apart metal–complex bonds and release free metal ions. These ions can then be removed through conventional methods, such as chemical precipitation, adsorption, coagulation, or ion exchange. Meanwhile, the AOPs also enable the oxidation of complexing agents into environmentally benign byproducts, such as water, CO_2_, and inorganic salts^183^ AOPs encompass a variety of techniques, including Fenton oxidation, ozonation, photocatalytic, ozonation photocatalytic oxidation, non-thermal plasma, UV/H_2_O_2_, as well as their combinations.^181,184,185^ These processes generate various reactive oxygen species (ROS), primarily the hydroxyl radical (˙OH), which acts as a non-selective and powerful oxidant for decomposing metal–ligand complexes (Fig. 6). The AOPs not only have the ability to degrade heavy metal complexes in water but also facilitate the recovery of heavy metals and decomposition of organic substances into water and carbon dioxide CO_2_.

The decomplexation of nickel, chromium, and copper EDTA complexes has been demonstrated to be effective through the use of systems such as pyrite/H_2_O_2_, cobalt/peroxymonosulfate (PMS), and persulfate/formate. This is achieved through the catalytic decomposition of hydrogen peroxide to produce ROS in Fenton and Fenton-like processes that are either homogeneous or heterogeneous.^186–191^ The use of PMS has proven successful in the decomplexation of Ni-EDTA in an alkaline solution, without the need for a catalyst.^192^ The utilization of various catalysts has enabled photocatalytic and electrocatalytic AOPs to exhibit exceptional efficiency in decomplexing heavy metals from industrial wastewater.^193–197^

The use of photocatalytic oxidation for the remediation of heavy metal complexes from wastewater has garnered significant attention as a research focus in recent years. The high oxidation capacity of photocatalysis allows for the effective destruction of heavy metal complexes, liberating free heavy metals, while simultaneously degrading and mineralizing organic ligands into water, carbon dioxide, and inorganic acids.^76,198^ The use of non-thermal plasma methods has recently gained considerable attention in the field of environmental remediation, particularly in the treatment of wastewater. This growing popularity is due to advantageous features such as the absence of chemical inputs, short processing time, compatibility for ambient conditions, high energy efficiency, environmental friendliness, and efficient degradation of recalcitrant organic contaminants.^199,200^ An investigation was conducted to evaluate the effectiveness of using discharge plasma in combination iron internal micro-electrolysis for the decomplexation of Cu-EDTA.^201^ The plasma oxidation was effective in destroying EDTA and the presence of iron played a key role in accelerating the decomposition process. A considerable number of studies published recently have focused on decomplexing copper-EDTA by means of non-thermal plasma oxidation, either alone or combined with alkaline precipitation.^202–207^ Zhu *et al.*^76^ discuss various methods for removing chelated heavy metals from wastewater, including their underlying mechanisms. The microwave-assisted Fenton reaction was shown to be effective in the rapid decomplexation of Ni-EDTA.^191^ While the majority of AOPs are still being tested in laboratory conditions, scaling them up for real-world applications is a crucial step that needs to be taken in the future.

## 4 Conclusions and future prospects

The pollution of aquatic systems by heavy metals, especially from sources such as industrial activities, chemical manufacturing, natural sources, and household usage, is a major concern in environmental protection. Human contamination is largely responsible for the widespread pollution of heavy metals in the environment, which is having a detrimental impact on human health. The long-term presence of heavy metals in the environment poses significant risks to both human health and ecosystems. Water contamination by these metals has become a major concern due to the accumulation of heavy metals in organisms, which pose a threat to human beings. The removal of heavy metals from wastewater has been achieved effectively through various treatment technologies, including physical adsorption using biochar, ion-exchange using zeolite, and the AOPs, which have received significant attention. This review focuses on the origins, toxicity, and recent advancements in the utilization of biochar-based adsorption, zeolite ion-exchange, and AOPs for the removal of heavy metals from wastewater. AOPs have the capacity to effectively recover heavy metals from heavy metal complexes. Moreover, zeolites, which are inexpensive adsorbent materials, possess a multitude of potential applications for the removal of heavy metals from wastewater. Studies have demonstrated that biochar-based adsorption exhibits exceptional results in removing heavy metal contaminants and is both environmentally friendly and sustainable. Developing effective methods for the recovery of heavy metals is crucial for the sustainable management of these valuable resources and reducing the environmental impact of heavy metal pollution. In-depth knowledge of biochar structure, physical and chemical properties, mechanism of removal and other relevant characteristics is crucial for developing effective methods for treating as-polluted waterways. Different analytical methods are being utilized to gain a better understanding of biochar. This review emphasizes the need for future research to focus on developing cost-effective and eco-friendly methods for removing heavy metals from wastewater. It also highlights the primary sources and health hazards of heavy metal contamination. In future studies, the aim should be to develop highly effective strategies that reduce heavy metal pollution while promoting sustainable economic development. A waste-to-resource approach should be considered as a means of removing harmful heavy metal ions from water systems. Further research is required to fully understand the mechanism and principles of heavy metal complex decomposition and heavy metal ion recovery using AOPs. To remove heavy metals effectively and efficiently, it is important to select environmentally friendly technology that can be scaled up for practical applications at a low cost.

## Author contributions

K. H. H. A.: conceptualization, data curation, investigation, resources, validation, supervision, visualization, writing – original draft, writing – review & editing. F. S. M.: software, visualization, writing – original draft, writing – review & editing. K. M. and S. H.: validation, writing – review & editing. R. F. H. and K. O. R.: writing – review & editing.

## Conflicts of interest

The authors declare no conflict of interest.

## Supplementary Material
